# Promoter Polymorphism in the *Serotonin Transporter (5-HTT)* Gene Is Significantly Associated with Leukocyte Telomere Length in Han Chinese

**DOI:** 10.1371/journal.pone.0094442

**Published:** 2014-04-07

**Authors:** Ping Li, Tiantian Liu, Jiajia Liu, Qing Zhang, Fenglan Lou, Feng Kong, Guanghui Cheng, Magnus Björkholm, Chengyun Zheng, Dawei Xu

**Affiliations:** 1 School of Nursing, Shandong University, Jinan, PR China; 2 Department of Microbiology, Shandong University School of Medicine, Jinan, PR China; 3 Central Research Laboratory, the Second Hospital, Shandong University, Jinan, PR China; 4 Hematology Department, the Second Hospital, Shandong University, Jinan, PR China; 5 Department of Medicine, Division of Hematology and Center for Molecular Medicine, Karolinska University Hospital Solna and Karolinska Institutet, Stockholm, Sweden; University of Birmingham, United Kingdom

## Abstract

The *serotonin transporter* gene (5-HTT)-linked polymorphic region (5-HTTLPR) plays an important role in modulating mood and behavior by regulating 5-HTT expression and thereby controlling the concentration of serotonin (5-HT) in brain synapses: The homozygous shorter allele (S/S) in 5-HTTLPR results in lower 5-HTT expression coupled with stronger psycho-pathological reactions to stressful experiences compared to the homozygous long (L/L) and heterozygous (S/L) alleles. Psychological insults and mood disorders have been shown to cause accelerated telomere shortening, a marker of biological aging, however, it is currently unclear whether the allelic variants of 5-HTTLPR affect telomere length (TL) in the healthy population without mood disorders. In the present study, we determined the relationship between TL and the 5-HTTLPR variants in healthy Han Chinese. The 5-HTTLPR genotyping and leukocyte TL analysis of 280 young female Han Chinese freshmen showed a significantly shorter TL in 149 of them carrying the 5-HTTLPR S/S version compared to those (131) with the L/S or L/S plus L/L genotypes (mean ± SD, 0.533±0.241 for S/S vs 0.607±0.312 for L/S, *P*  =  0.034; or vs 0.604±0.313 for L/S plus L/L, *P*  =  0.038). Similar results were achieved in the other cohort including 220 adult healthy individuals of different age, gender and profession (0.691±0.168 for S/S vs 0.729±0.211 for L/S, *P*  =  0.046, or vs 0.725±0.213 for L/S plus L/L, *P*  =  0.039). Taken together, shorter leukocyte TL is significantly associated with the 5-HTTLPR S/S allelic variant, which may be implicated in psychological stress-related health problems.

## Introduction

Telomeres, tandem arrays of repetitive TTAGGG sequences associated with their binding factors, form protective caps at chromosome termini and are essential to maintain genomic integrity and stability [1], [2]. Telomeric DNA is synthesized by telomerase, an RNA-dependent DNA polymerase [2], [3]. In human somatic cells, telomeres are 8 to 20 kb long and shorten progressively with each round of cell division or with increased age owing to “the end replication problem” and lack of telomerase activity [1]–[3]. When telomeres become too short (dysfunctional) to protect chromosomes, the DNA damage response is activated, thereby triggering the permanent growth arrest of cells (replicative senescence) [1], [2], [4]. Recent evidence has accumulated that the same scenario also occurs *in vivo*, thereby contributing to human aging [3], [4]. Moreover, it has been shown that individuals with shorter leukocyte telomeres, exhibit a higher risk to develop age-related diseases such as heart diseases, stroke and cancer [1]–[4]. More importantly, a close correlation between shorter telomeres and increased mortality has been documented in published reports [5]–[8].

In addition to the cell replication- and aging-mediated telomere shortening, many other factors may significantly affect telomere length (TL). For instance, an impact of psychosocical factors and mood disorders on TL has been observed. In 2004, Epel et al [9] first reported significantly shorter telomeres in leukocytes derived from women experiencing high levels of life stress, and they thus suggested the accelerated telomere erosion as a link between psychological stress and early onset of age-related diseases. Since then, the relationship between TL and different kinds of psychological adversity has been extensively investigated, and most of the study results consistently confirmed this inverse relationship [10]–[13].

The monoamine neurotransmitter serotonin or 5′-hydroxytryptamine (5-HT) is involved in the regulation of diverse brain functions including emotional and behavioral activities through interactions with different 5-HT receptor subtypes in the central nervous system (CNS) [14]. Appropriate levels of 5-HT at brain synapses is essential for these functions and one key regulator of 5-HT is serotonin transporter (5-HTT) on the presynaptic neuron [14]. 5-HTT removes 5-HT released into the synaptic cleft, thereby terminating serotonergic neurotransmission. 5-HTT protein is encoded by the *SLC6A4* gene whose transcriptional activity is regulated by a number of variations [14]. Among these, the *serotonin transporter* gene (5-HTT)-linked polymorphic region (5-HTTLPR), composed of short (S/S), or long (L/L) homozygous, or heterozygous (S/L) allelic versions, has been well characterized to affect 5-HTT expression: The S and L alleles in the 5-HTTLPR result in lower and higher levels of 5-HTT expression, respectively [14], [15]. People carrying the S/S version in general exhibit lower expression of 5-HTT coupled with reduced reuptake of 5-HT from the synapse, leading to stronger psycho-pathological reactions to stressful experiences compared to those with the L/L or L/S allele [16]. Numerous observations have also suggested a positive correlation between the S/S genetic phenotype and anxiety-related personality and susceptibility to mood disorders [17]–[24]. Given the well established association between shorter telomeres and psychosocial insults discussed above, we hypothesize that the 5-HTTLPR variants may affect TL in the general population without mood disorders. In the present study, we address this issue by analyzing the relationship between leukocyte TL and 5-HTTLPR genotypes in two cohorts of adult healthy individuals.

## Results

### 5-HTTLPR variant distribution in the two cohorts of healthy adults

**Figure 1:**
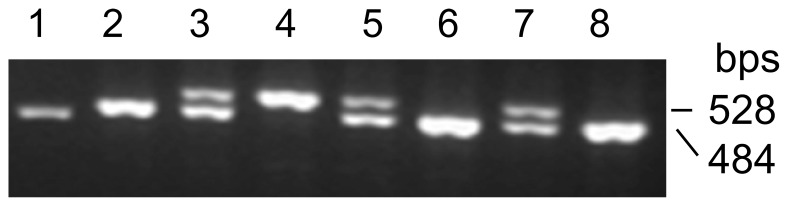
The 5-HTTLPR genotyping by PCR. The 5-HTTLPR is located in the promoter region of the gene 1.2 kb upstream of the transcriptional start site. S and L variants contain 14 and 16 repeats, respectively (22 bps/repeat). The specific PCR primers span the LPR region and genomic DNA derived from 500 healthy individuals was amplified. Shown are the representative S/S, L/S and L/L genotypes. SS: Lanes 2, 6 and 8; LS: Lanes 3, 5 and 7; LL: Lane 4. Lane 1: DNA marker.

The 5-HTTLPR genotyping was performed using polymerase chain reaction (PCR) and the representative images are shown in Fig. 1. The analysis results obtained from 280 female freshmen are listed in Table 1, and among them, individuals carrying the S/S, L/S and L/L variants were 149 (53.2%), 105 (37.5%) and 26 (9.3%), respectively. The detailed variant distribution in each age group is shown in Table 1. The distribution of the 5-HTTLPR variant differed somewhat in the other cohort of 220 adult participants, with S/S, L/S and L/L carriers being 105 (47.7%), 100 (45.5%) and 15 (6.8%), respectively. Tables 2 and 3 provide each variant according to gender and age groups.

**Table 1:** 5-HTTLPR genotype distribution of each age group among 280 female freshmen.

Age (years)	S/S (%)	L/S (%)	L/L (%)	Total
16	1 (100)	0 (0)	0 (0)	1
17	13 (44.8)	9 (31.0)	7 (24.2)	29
18	71 (54.2)	46 (35.1)	14 (10.7)	131
19	55 (57.3)	38 (39.6)	3 (3.1)	96
20	8 (42.1)	10 (52.6)	1 (5.3)	19
21	1 (25.0)	2 (50.0)	1 (25.0)	4
Total	149 (53.2)	105 (37.5)	26 (9.3)	280

**Table 2:** 5-HTTLPR genotype distribution of each age group among 220 healthy individuals.

Age (years)	S/S (%)	L/S (%)	L/L (%)	Total
21 – 29	27 (52.9)	22 (43.1)	2 (4.0)	51
30 – 39	26 (47.3)	24 (43.6)	5 (9.1)	55
40 – 49	21 (58.3)	14 (38.9)	1 (2.8)	36
50 – 59	18 (42.9)	22 (52.4)	2 (4.7)	42
60 – 69	4 (57.1)	2 (28.6)	1 (14.3)	7
70 – 79	7 (30.4)	13 (56.5)	3 (13.1)	23
80 – 85	2 (33.3)	3 (50.0)	1 (16.7)	6
Total	105 (47.7)	100 (45.5)	15 (6.8)	220

**Table 3:** 5-HTTLPR variant distribution between male and female individuals.

Gender	S/S (%)	L/S (%)	L/L (%)	Total
Male	53 (43.3)	58 (47.5)	11 (9.1)	122
Female	52 (53.0)	42 (42.9)	4 (4.1)	98
Total	105 (47.7)	100 (45.5)	15 (6.8)	220

### Significantly shorter telomeres in the individuals carrying the 5-HTTLPR S/S variant

Leukocyte TL was determined using quantitative real-time PCR (qPCR). In the cohort of 280 freshmen aged 16 to 21 years, there was no correlation between age and TL (data not shown). The relative TL (mean ± SD) was 0.533±0.241, 0.607±0.312 and 0.591±0.319 for the S/S, L/S and L/L carriers, respectively (Table 4), and a statistically significant difference was found between S/S and L/S groups (*P*  =  0.034). A similar result was obtained when the comparison was made between S/S and L/S plus L/L group (0.533±0.241 vs 0.604±0.313, *P*  =  0.038) (Table 4). However, there was no difference in TL between the S/S and L/L groups, largely due to too few L/L carriers (26 individuals) in this cohort. In addition, TL of L/S and L/L carriers did not differ.

**Table 4:** Difference in telomere length among different 5-HTTLPR genotypes.

Genotype	N	TL* (mean ± SD)	*P* value
Freshmen cohort			
S/S	149	0.533 ± 0.241	0.034 (S/S vs L/S)
L/S	105	0.607 ± 0.312	>0.05 (L/S vs L/L)
L/L	26	0.591 ± 0.319	>0.05 (L/L vs S/S)
L/S + L/L	131	0.604 ± 0.313	0.038 (S/S vs L/S + L/L)
Second cohort			
S/S	105	0.691 ± 0.168	0.046 (S/S vs L/S)
L/S	100	0.729 ± 0.211	>0.05 (L/S vs L/L)
L/L	15	0.697 ± 0.235	>0.05 (L/L vs S/S)
L/S + L/L	115	0.725 ± 0.213	0.039 (S/S vs L/S + L/L)

*TL: Telomere length.

**Figure 2:**
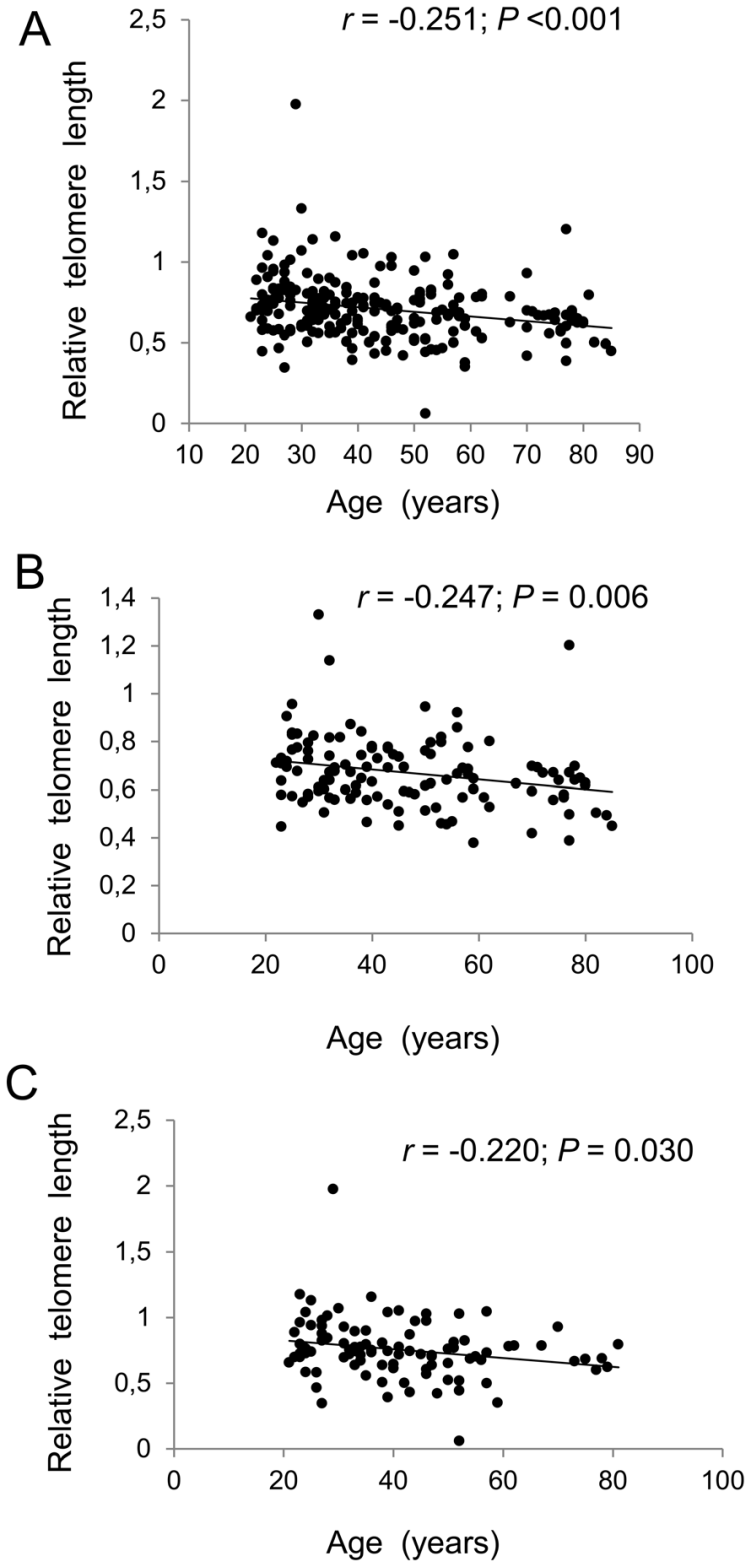
Age-related telomere shortening in 220 healthy adults. TL was determined using qPCR and expressed as arbitrary units. (A) Total 220 individuals. (B) Ninety-eight females. (C) One hundred and twenty-two males.

To verify the result obtained from 280 young female freshmen, we further recruited another cohort of 220 adult individuals of different age, gender and profession. As expected, there was a significant correlation between age and TL in the whole cohort (*r*  =  −0.251, *P*<0.001), and the age-dependency of TL was still seen when male and female groups were analyzed separately (Fig. 2). We then compared differences between different 5-HTTLPR genotypes. As shown in Table 4, the S/S carriers exhibited significantly shorter TL than that of the L/S individuals (0.691±0.168 vs 0.729±0.211, *P*  =  0.046), after adjusting for age. When the L/S and L/L carriers were pooled together, this group had a significantly longer TL than S/S carriers (0.725±0.213 vs 0.691±0.168, *P*  =  0.038) (Table 4). Once again, there were no differences in TL between the S/S and L/L carriers, mainly because of too few of individuals (15) in the latter group.

## Discussion

In the present study, we explored the relationship between TL and 5-HTTLPR genotypes by studying two cohorts of 500 healthy Han Chinese adults. In 280 young female freshmen, we observed a significantly shorter leukocyte TL in the homozygous S/S carriers compared to those harboring heterozygous L/S or a combined group including both L/S and homozygous L/L individuals. This correlation was further verified in another cohort of 220 healthy adults of different age, gender and profession. The findings collectively suggest that the healthy individuals carrying the S/S 5-HTTLPR variant undergo accelerated telomere erosion.

Previous studies have shown that perceived stress and different psychological insults are associated with an increased rate of telomere shortening, suggesting a strong psychological impact on telomere homeostasis [9]–[11]. All of the healthy individuals included in the present study were free of mood disorders, however, there has long been a link of the neurobehavioral effects of the 5-HTTLPR s-variant with hypervigilance, an enhanced sensitivity to motivationally relevant environmental stimuli [14]. For example, they exhibit exaggerated reactivity in the amygdala and increased startle responses to pictures of fearful faces, a stronger attentional bias for negatively valenced words, difficulty with disengaging attention from threat-related stimuli, increased fear responses to a shockpredicting stimulus, and so on [17]–[24]. Alternatively, the S/S carriers may display much lower threshold to the above stimuli, and the continuous accumulation of all these emotional responses mimics psychological stress, thus leading to telomere shortening. Oxidative stress, inflammation and lower telomerase activity have been implicated in the psychological insults and mood disorders-mediated telomere shortening [25]–[27], however, exact mechanisms remain to be defined, and we are currently addressing these issues.

The age range was from 16 to 21 years in the cohort of 280 freshmen, and the S/S variant effect on TL was already seen at that age in their leukocytes, which indicates that this event might occur early in their life. A previous study demonstrated that middle-aged UK women who experienced more stressful events (such as physical abuse, parental divorce, unemployment or drug use) during their childhood, had shorter telomeres [28]. Likely, telomere homeostasis is vulnerable to various negative factors including psychological insults in childhood because of physiologically rapid telomere erosion in this period [29]. Of note, the TL difference between the 5-HTTLPR S/S and L/S variant carriers was smaller in the second cohort of individuals (5.5%) than in the freshmen one (13.9%). Because the second cohort included more senior adults, it would be interesting to probe whether senior S/S carriers may become less sensitive to various environmental stimuli, and re-gain net TL to a certain degree.

In the initial pilot study, we selected a relatively homogenous cohort of individuals, young female Chinese Han freshmen, for the following reasons: First, TL is highly variable within individuals and among different races [30]–[32]. Second, TL is equal between the sexes at birth, but age-mediated telomere shortening occurs more rapidly in males than in females [33]. Third, TL is affected by many lifestyles and environmental factors, such as smoking, night-shift work, and certain professions [25], [34]. By making this selection, we wanted to minimize other effects on TL in the studied subjects. Therefore, the observed differential TL may reflect true difference between the 5-HTTLPR S/S and L/S-L/L carriers. Further support for such a difference comes from the other cohort of 220 adult individuals with different age, gender and profession.

It is well established that the presence of shorter telomeres is a feature of many age-related conditions and diseases including immunosenescence, cardiovascular disease, stroke, cancer, sarcopenia, osteoporosis, osteoarthritis, and skin aging [1], [4], [5]–[8], [35]–[40]. Therefore, the observed shorter telomere among individuals experiencing psychological insults may provide an explanation for early demonstrations of psychopathological conditions-mediated premature onset of aging. It is currently unclear whether 5-HTTLPR S/S carriers have a higher risk to develop aging-related diseases. Given the present finding, it is conceivable that this may happen and is thus worth of evaluating such possibility.

We identified 50.8% S/S, 41.0% L/S and 8.2% L/L for 5-HTTLPR variants in these 500 Chinese Han healthy adults. The total frequency for L and S alleles is 28.7% and 71.3%, respectively, which is rather different from the variant distribution observed in Europeans (57% for L allele; 95% CI: 49.9–61.8%), however, consistent with the results obtained from Asians (27% for L allele; 95% CI: 23.9–32.9%) [16]. It is unclear whether there is a negative correlation between the 5-HTTLPR S/S genotype and reduced TL in the European population, or whether such differential distribution of the 5-HTTLPR variants influences TL between eastern and western worlds, thereby contributing to the differential frequencies of aging-related diseases. Further studies are required to address these important issues.

In summary, our present study shows the accelerated telomere attrition in the Chinese Han 5-HTTLPR S/S variant carriers, however, the underlying mechanism is unclear. Given the accumulated evidence that the 5-HTTLPR S/S variant is strongly associated with the enhanced sensitivity to motivationally relevant environmental stimuli, we are currently investigating whether the S/S genotype mimics adverse psychological insults, thereby resulting in reduced TL.

## Materials and Methods

### Participants and sample collection

The study included two cohorts of Chinese Han healthy individuals, one with 280 young female freshmen enrolled in Shandong University in 2012 and the other with 220 adult healthy volunteers of different professions. Both cohorts of participants were free from depression or mood disorders, and did not take statins, estrogens, or other medicines regularly according to their medical history. Age of freshmen was from 16 to 21 years (median age 18 years). The cohort of 220 adults included 98 females and 122 males. The median age of females was 38 years (range 21 to 81 years). Men had median age of 43 years (range 22 to 85 years) (For details see Tables 1 and 2). Peripheral blood was collected in the morning before breakfast, leukocytes were isolated with red blood cell lysis buffer (Tiangen, China) and the cells were then used immediately or stored at −80°C. The study was approved by the ethics committee and review board of Shandong University Nursing School. The oral informal consent, which is consistent with the institutional regulation and approved by the ethics committee of Shandong University Nursing School, was obtained from the participants or guardians if <18 years and documented in the study subject list. Blood was collected only from those who agreed.

### DNA extraction and 5-HTTLPR genotyping

Genomic DNA was extracted from participants' peripheral blood leukocytes using a DNA extraction kit (TianGen, China). The analysis of the 5-HTTLPR variant was done using PCR. The following primer pair was designed to span the variant region in the *5-HTT* promoter: 5′-GGCGTTGCCGCTCTGAATGC-3′ (forward) and 5′-GAGGGACTGAGCTGGACAACCAC-3′ (reverse). PCR was performed with the annealing temperature at 62°C for 33 cycles. The PCR products were subjected to electrophoresis in 2% agarose gels, stained with ethidium bromide and visualized under UV light.

### Telomere length assessment

TL was determined using qPCR as described [41], [42]. Two ng of DNA were used for each PCR reaction and PCR was carried out in an ABI7700 sequence detector (Applied Biosystems, Foster City, CA). The primer sequences for human telomere (Tel 1b and Tel 2b) and β-globin (HBG3 and HBG4) were: Tel1b: 5′-CGGTTTGTTTGGGTTTGGGT-TTGGGTTTGGGTTTGGGTT-3′; Tel2b: 5′-GGCTTGCCTTACCCTTACCCTTACCC-TTACCCTTACCCT-3′; HBG3: 5′-TGTGCTGGCCCATCACTTTG-3′, and HBG4: 5′-ACCAGCCA-CCACTTTCTGATAGG-3′. T/HBG values were determined using the formula T/S  =  2^−ΔCt^, where ΔCt  =  average Ct_telomere_ − average Ct_β-globin._ The T/S ratio was arbitrarily expressed as TL.

### Statistical analyses

Differences in TL between different 5-HTTLPR genotypes were analyzed using an analysis of ANCOVA, among which age and/or gender as covariates were corrected for TL. The relationship between TL and age was determined using Pearson's correlation analysis. All the tests were two-tailed and computed using SigmaStat3.1 software (Systat Software, Inc., Richmond, CA). *P* values of <0.05 were regarded as statistically significant.
